# Insights into the Interaction Mechanisms of Peptide and Non-Peptide Inhibitors with MDM2 Using Gaussian-Accelerated Molecular Dynamics Simulations and Deep Learning

**DOI:** 10.3390/molecules29143377

**Published:** 2024-07-18

**Authors:** Wanchun Yang, Jian Wang, Lu Zhao, Jianzhong Chen

**Affiliations:** School of Science, Shandong Jiaotong University, Jinan 250357, China; wangjian_lxy@sdjtu.edu.cn (J.W.); zhaolusdu@163.com (L.Z.)

**Keywords:** MDM2, peptide inhibitors, Gaussian-accelerated dynamics simulations, deep learning, binding free energy

## Abstract

Inhibiting MDM2-p53 interaction is considered an efficient mode of cancer treatment. In our current study, Gaussian-accelerated molecular dynamics (GaMD), deep learning (DL), and binding free energy calculations were combined together to probe the binding mechanism of non-peptide inhibitors K23 and 0Y7 and peptide ones PDI6W and PDI to MDM2. The GaMD trajectory-based DL approach successfully identified significant functional domains, predominantly located at the helixes α2 and α2’, as well as the β-strands and loops between α2 and α2’. The post-processing analysis of the GaMD simulations indicated that inhibitor binding highly influences the structural flexibility and collective motions of MDM2. Calculations of molecular mechanics–generalized Born surface area (MM-GBSA) and solvated interaction energy (SIE) not only suggest that the ranking of the calculated binding free energies is in agreement with that of the experimental results, but also verify that van der Walls interactions are the primary forces responsible for inhibitor–MDM2 binding. Our findings also indicate that peptide inhibitors yield more interaction contacts with MDM2 compared to non-peptide inhibitors. Principal component analysis (PCA) and free energy landscape (FEL) analysis indicated that the piperidinone inhibitor 0Y7 shows the most pronounced impact on the free energy profiles of MDM2, with the piperidinone inhibitor demonstrating higher fluctuation amplitudes along primary eigenvectors. The hot spots of MDM2 revealed by residue-based free energy estimation provide target sites for drug design toward MDM2. This study is expected to provide useful theoretical aid for the development of selective inhibitors of MDM2 family members.

## 1. Introduction

The tumor suppressor protein p53 plays a crucial role in regulating the cell cycle, promoting apoptosis, and repairing DNA damage, thereby safeguarding cells against malignant transformation [1,2]. The active form of p53 is highly effective in suppressing the development of tumors. Notably, approximately 50% of all human cancers exhibit malfunctions in p53 function due to deletions or mutations in its DNA-binding domain [3]. A host of strategies targeting the p53 pathway have emerged due to its inhibitory effects against tumors [4,5]. Numerous proteins play critical roles in regulating the function of p53, such as MDM2 [6,7,8,9,10] and MDMX [11,12,13]. In fact, inactivated p53 fails to yield appropriate responses to external stimuli, substantially increasing the risk of tumorigenesis. The overexpression of MDM2/MDMX and p53-MDM2 interactions leads to the inactivation of p53, controlled by negative feedback mechanisms or p53-MDM2. Thus, it is of high significance to probe effective approaches to hold back the MDM2-p53 interaction.

**Figure 1:**
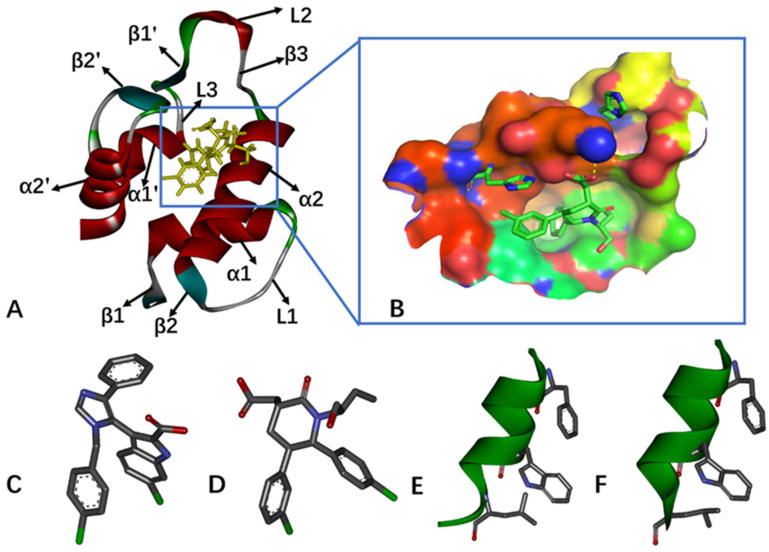
Molecular structures: (**A**) inhibitor-bound MDM2, in which secondary structures are labeled; (**B**) binding pocket of MDM2 protein; (**C**) K23; (**D**) 0Y7; (**E**) PDI6W; and (**F**) PDI.

Despite significant sequence variations, all MDM2 proteins exhibit a consistent topological structure featuring a left-handed bundle of four alpha helixes (α1, α2, α1’, α2’), interconnected by β-strands and loops that dictate substrate specificity [14,15]. The secondary structures of MDM2 bound to the inhibitor and the binding pocket are illustrated in Figure 1A,B, respectively. The hydrophobic sidechains of residues Phe19’, Trp23’, and Leu26’ from p53 play a crucial role in mediating the interaction between p53 and MDM2 [16,17,18]. These residues directly disrupt the binding of p53 to MDM2, making them a promising target for potential anticancer therapies. Various drug candidates, including small-molecule inhibitors and peptide inhibitors, have been developed to target the p53-MDM2 interaction. Peptide inhibitors that disrupt the interactions between p53 and its negative regulator MDM2 have the potential to activate p53 [19,20,21,22,23]. Additionally, lots of small-molecule inhibitors have been designed using structure-based approaches [24,25,26,27,28,29]. Some of these inhibitors are undergoing clinical evaluation for anticancer treatment [30,31,32,33,34]. There is ongoing research focused on developing potent inhibitors that target the MDM2–p53 interaction.

Enhancing our understanding of how these inhibitors bind to MDM2 at atomic levels is helpful for the development of potent inhibitors that disrupt the MDM2–p53 interaction, and also provides crucial insight into the structure–affinity relationships of MDM2–inhibitor complexes. Various tools are currently available to probe the conformational dynamics of targets, such as conventional molecular dynamics (cMD) [35,36,37,38,39], Gaussian-accelerated molecular dynamics (GaMD) simulations [21,40,41,42,43], and calculations of binding free energies [44,45,46,47]. These methods have been extensively utilized to uncover the molecular mechanisms and free energy bases of target–ligand identification [48,49,50,51,52,53]. GaMD simulations, particularly, effectively overcoming energy barriers in protein systems, have shown successes in studying changes in conformational dynamics and the free energy profiles of targets. To extract valuable information from cMD or GaMD trajectories, MD simulations and machine learning (ML) have been used to deepen our understanding of the functions of targets and ligand–target binding mechanisms [54,55,56,57,58]. Miao’s group developed a GaMD trajectory-based deep learning (DL) approach called the GaMD, DL, and free energy profiling workflow (GLOW) to effectively decode the molecular mechanisms related to GPCR activation and allosteric modulation [59,60]. Furthermore, the GaMD trajectory-based DL approach also obtained successes in the ligand–target identification of molecular mechanisms and the exploration of significant structural domains of targets [61,62]. Binding free energy calculations are powerful tools for elucidating the interaction mechanisms between inhibitors and their targets. The MM-GBSA and SIE methods have been identified as effective approaches for calculating the binding free energies of inhibitors to proteins [63,64,65,66]. They have been successfully applied to elucidate protein–protein and protein–inhibitor interactions.

In this study, we focus on investigating the binding mechanisms of four inhibitors to MDM2, including two non-peptide inhibitors K23 and 0Y7, and peptide inhibitors PDI6W and PDI. Regarding the two non-peptide inhibitors, K23 and 0Y7, K23 comprises four aromatic groups, efficiently occupying the binding pockets of MDM2 with a median inhibitory concentration (IC50) value of 1.71 μM [67], while 0Y7 is a piperidinone inhibitor that interacts favorably with the N-terminus of human MDM2, with an IC50 value of 50 nM [68]. With respect to the two peptide inhibitors, PDI6W and PDI, PDI6W has a residue sequence (LTFEHWWAQLTS) with an IC50 value of 36 nM toward MDM2 [22]; the other peptide inhibitor, PDI, possesses a residue sequence (LTFEHYWAQLTS) showing inhibiting ability on MDM2 with an IC50 of 44 nM [23]. The structures of these inhibitors are depicted in Figure 1C–F. Multiple independent Gaussian-accelerated molecular dynamics (MI-GaMD) simulations were conducted to improve conformational sampling, and deep learning (DL) was utilized to identify critical structural domains. Additionally, principal component analysis (PCA) [69,70,71,72] and the construction of free energy landscapes (FELs) were performed to explore conformational spaces and the free energy profile of MDM2. Calculations of binding free energies and energy decomposition analysis were conducted to evaluate the binding difference between the two types of inhibitors of MDM2 and identify hot spots of MDM2 inhibitors. We expect that this work can offer valuable insights into the mechanisms underlying the inhibition of the p53-MDM2 interaction.

## 2. Results and Discussion

### 2.1. Differences in the Contacts of Structural Domains Revealed by Deep Learning

**Figure 2:**
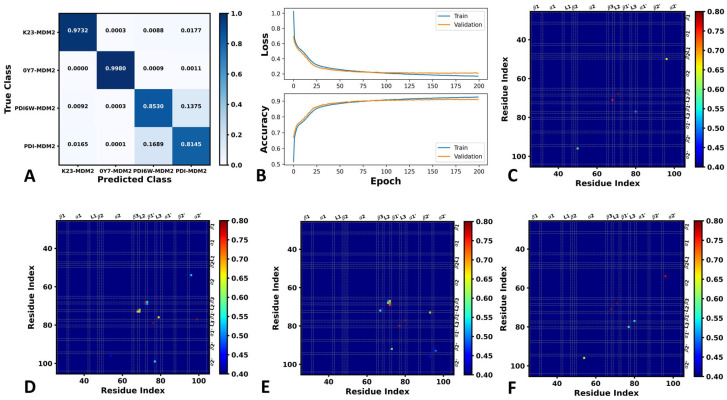
Classification and saliency map of residue contact gradients: (**A**) classification of K23-MDM2, 0Y7-MDM2, PDI6W-MDM2 and PDI-MDM2; (**B**) learning curves of the training and validation datasets; and (**C**–**F**) the saliency map of residue contact gradients for K23-MDM2, 0Y7-MDM2, PDI6W-MDM2 and PDI-MDM2.

The DL procedure involved several steps: (1) the conformations extracted from the GaMD trajectories were converted into images suitable for DL analysis; (2) these images were randomly split into a training set and a validation set to conduct DL; (3) the outcomes of the DL training were visualized, as shown in Figure 2. Figure 2A shows the classification information and Figure 2B depicts the learning curves of the training and validation datasets.

For the inhibitor-bound MDM2, the overall accuracy reached 91.12% in the validation set after 200 epochs (Figure 2B). Using 8000 snapshots for the validation of each system, four systems were correctly identified (Figure 2A). Specifically, 7786 snapshots of K23-MDM2, 7894 snapshots of 0Y7-MDM2, 6824 snapshots of PDI6W-MDM2, and 6515 snapshots of PDI-MDM2 were accurately recognized. However, there were instances of misidentification. In detail, 142, 70, and 2 snapshots from 8000 of K23-MDM2 were inaccurately classified as PDI-MDM2, PDI6W-MDM2, and 0Y7-MDM2, respectively. Similarly, 9 and 7 snapshots from 8000 of 0Y7-MDM2 were erroneously categorized as PDI-MDM2 and PDI6W-MDM2, respectively. Furthermore, 1100, 74, and 2 snapshots from 8000 of PDI6W-MDM2 were misidentified as PDI-MDM2, K23-MDM2, and 0Y7-MDM2, respectively. Additionally, 1351, 132, and 1 snapshots from 8000 of PDI-MDM2 were mistakenly recognized as PDI6W-MDM2, K23-MDM2, and 0Y7-MDM2, respectively. It is observed that a higher inaccuracy of identification occurs between two peptide inhibitors, which may be due to the highly similar binding modes of PDI6W and PDI to MDM2. 

The pixel-attributed residue contact gradient maps of the most populated MDM2 structures are depicted in Figure 2C–F. Overall, the characteristic residue contacts of K23-MDM2 are situated between helix α2 and α2’, β-turn β3 and loop L2, and β-strand β1’and loop L3. Similarly, the characteristic residue contacts of 0Y7-MDM2 are found between helix α2 and α2’, β-turn β3 and loop L2, β-turn β3 and β-strand β1’, β-strand β1’ and loop L3, and β-strand β1’ and helix α2’. In comparison to K23-MDM2, 0Y7-MDM2 reveals new contacts between β3 and β1’, as well as β1’ and α2’. 

The characteristic residue contacts of PDI6W-MDM2 are observed between helix α2’ and β-strand β2’, β-turn β3 and loop L2, β-strand β1’ and loop L3, and β-strand β1’ and β2’. Meanwhile, the characteristic residue contacts of PDI-MDM2 are noted between helix α2 and α2’, β-turn β3 and loop L2, and β-strand β1’ and loop L3. In contrast to PDI6W-MDM2, PDI-MDM2 introduces new contacts between α2 and α2’, losing contacts between α2’ and β2’, and β1’ and β2’. Prior experimental studies have highlighted the significance of α2, α2’, β3, L2, β1’, L3, and β2’ in the binding of inhibitors to MDM2 [22,23,67,68], aligning with our current findings.

### 2.2. Free Energy Profiles and Structural Dynamics of MDM2

To gain insights into the dynamic behavior of MDM2, PCA was conducted on the GaMD trajectories of inhibitor-bound MDM2, with the Cα atom coordinates saved in the single GaMD trajectory (SGT) integrated by using three independent GaMD trajectories. The function of eigenvalues over eigenvector indexes was estimated (Figure S1). An eigenvalue is commonly employed to characterize the structural fluctuations of proteins along an eigenvector. The first six eigenvalues accounted for 57.27, 67.96, 56.12, and 51.23% of the total movements of the K23-, 0Y7-, PDI6W-, and PDI-bound MDM2 structures, respectively. Notably, the first eigenvalue of 0Y7-MDM2 is much greater than that of the other inhibitor-bound MDM2 structures, suggesting a greater structural fluctuation amplitude of the 0Y7-bound MDM2 structure along the primary eigenvectors relative to the three other complexes (Figure S1).

**Figure 3:**
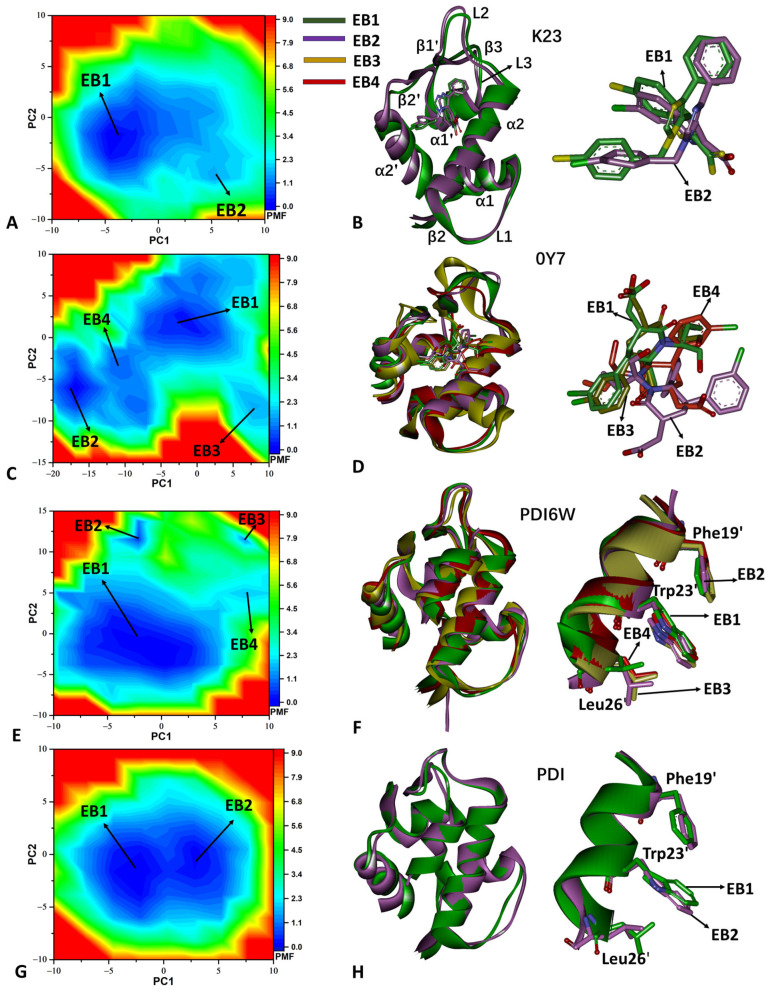
Free energy profiles and representative structures of inhibitor-bound MDM2: (**A**,**C**,**E**,**G**) correspond to the FELs of K23-MDM2, 0Y7-MDM2, PDI6W-MDM2, and PDI-MDM2, respectively; (**B**,**D**,**F**,**H**) indicate the superimposition of representative structures of MDM2 and inhibitors trapped in different EBs. The free energy is scaled in kcal/mol.

To analyze the variations in the free energy profiles of MDM2, SGTs were projected onto the first two eigenvectors, and these projections were used as the reaction coordinates (RCs) to construct FELs, which are illustrated in Figure 3. The GaMD simulations recognized two, four, four, and two energy basins (EBs) in the K23-, 0Y7-, PDI6W-, and PDI-bound MDM2 structures (Figure 3A,C,E,G), respectively, with 0Y7 showing the most pronounced impact on the free energy profiles of MDM2. To investigate the structural variances within different EBs, the representative structures falling into the EBs were superimposed together (Figure 3B,D,F,H). The β-strands and loops located between α2 and α1’ of the 0Y7-bound MDM2 structure, including β3, L2, β1’, and L3, exhibited the most highly obvious deviation among the various energy states; moreover, this deviation induced changes in the binding poses of 0Y7. It is observed that 0Y7 has four different binding poses (Figure 3D). Although the structures of K23-bound MDM2 in EB1 and EB2 are aligned well, the binding poses of K23 yield sliding and rotation, which induces two different binding orientations (Figure 3B). In spite of the four energy basins (EB1–EB4) of PDI6W-bound MDM2, their representative structures hardly produce obvious deviations; moreover, three key residues (Phe19’, Trp23’, and Leu26’) in PDI6W are also aligned well (Figure 3F). In the PDI-MDM2 complexes, loop L1 deviates between the presentative structures EB1 and EB2; correspondingly, two key residues, Trp23’ and Leu26’ in PDI, generate obvious sliding (Figure 3H). The aforementioned changes certainly impact the binding of the peptide and non-peptide inhibitors to MDM2.

**Figure 4:**
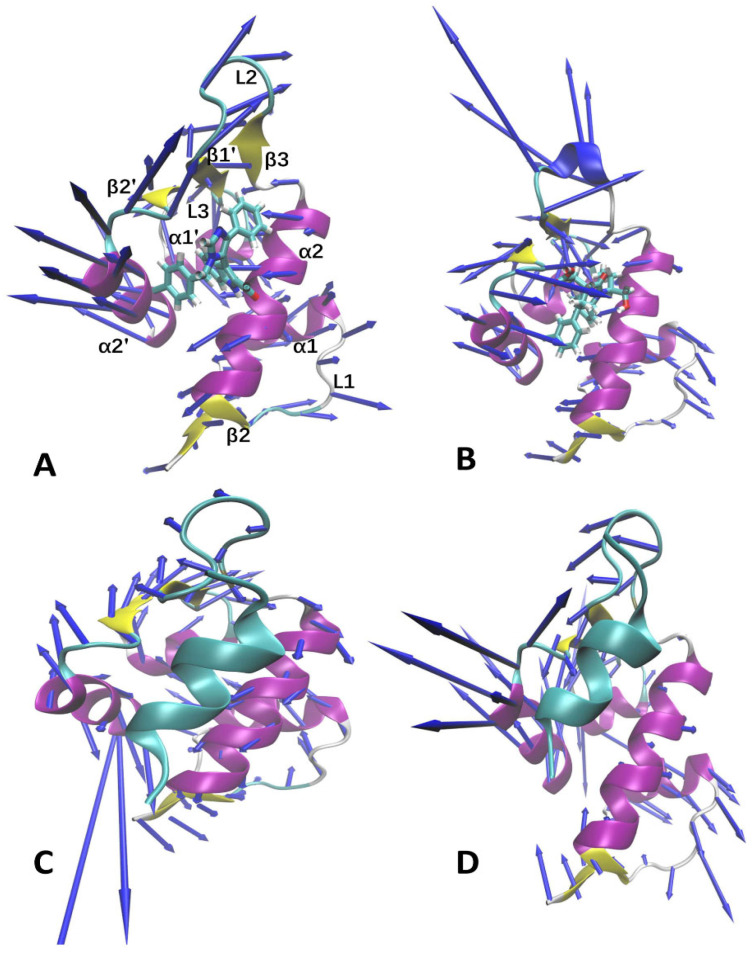
Concerted motions of MDM2 revealed by the first eigenvector via PCA: (**A**) K23-MDM2, (**B**) 0Y7-MDM2, (**C**) PDI6W-MDM2, and (**D**) PDI-MDM2.

In order to probe the effects of the two types of inhibitors on the collective motions of MDM2, the first eigenvector was visualized using the VMD program [73] and the results are depicted in Figure 4. Except for the N- and C-terminals of MDM2, the binding of peptide and non-peptide inhibitors has an obvious effect on the conformations of loops L2 and L1 from MDM2. Compared to the two non-peptide inhibitors (Figure 4A,B), the binding of the two peptide inhibitors, PDI6W and PDI, inhibits the collective motions of loop L2, which implies that these two peptide inhibitors possibly yield interactions with L2, β1’, and β3 (Figure 4C,D). In comparison with K23 and 0Y7, the binding of PDI6W changes the fluctuation tendency of loop L1 from MDM2 along the first eigenvector, but the binding of PDI hardly affects the concerted motions of this loop (Figure 4D). In addition, the concerted motions of helix α2’ are also affected by inhibitor binding (Figure 4).

Based on the above information, the binding of peptide and non-peptide inhibitors exerts different influences on the free energy profiles of MDM2, the binding poses of inhibitors, and the concerted motions of structural domains. The structural information revealed by the PCA and FELs is in basic agreement with the results from the DL approach. Meanwhile, our current findings are also consistent with the previous results revealed by MD simulations [37,38]. According to their structural information, peptide and non-peptide inhibitors bind to the hydrophobic cleft of MDM2 and occupy the binding position of p53 in MDM2, which not only leads to conformational changes in the binding cleft of MDM2, but also effectively prevents p53 from going into the binding pocket of MDM2. The changes in the concerted motions of the structural domains and free energy profiles due to the presence of the two types of inhibitors can impact inhibitor–MDM2 binding.

### 2.3. Structural Property of MDM2

**Figure 5:**
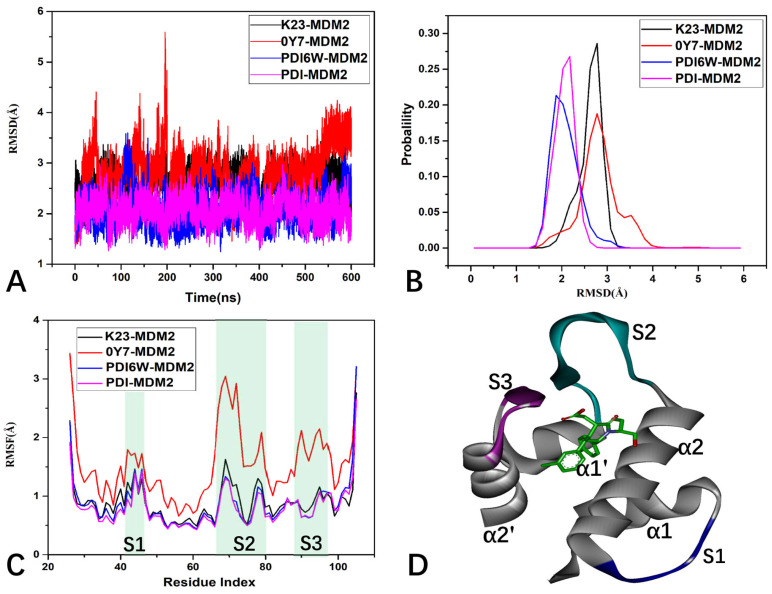
The RMSD and RMSF values of MDM2 in GaMD simulations: (**A**) the time course of RMSDs for MDM2; (**B**) the probability distribution of RMSDs for MDM2; (**C**) the RMSFs of the Cα atoms from MDM2; and (**D**) the flexibility domains revealed by GaMD simulations.

To investigate the structural stability of MDM2, root-mean-square deviations (RMSDs) of backbone atoms from MDM2 were computed throughout the GaMD simulations, with reference to the initially optimized structures (Figure 5A,B). The time course of the RMSDs indicates that the structures of MDM2 in the four systems are stable (Figure 5A). The distributions of RMSDs are estimated in Figure 5B. For the two non-peptide inhibitors, the RMSD of K23-MDM2 is distributed at ~2.75 Å, and that of 0Y7-MDM2 at the peaks of 2.75 and 3.43 Å (Figure 5B). As for the two peptide inhibitors, the RMSDs of PDI6W-MDM2 and PDI-MDM2 are populated at the peaks of 1.61 and 2.98 Å, respectively (Figure 5B). Compared to the two non-peptide inhibitors, the binding of peptide inhibitors decreases the RMSDs of MDM2. This result suggests that the binding of peptide inhibitors is more favorable for the structural stability of MDM2 than non-peptide inhibitors, implying that PDI6W and PDI should generate more interaction contacts with MDM2 than K23 and 0Y7. Meanwhile, our results also show that the GaMD trajectories of the four complexes are reliable for the post-processing analyses.

To understand the inhibitor-mediated effect on structure flexibility, root-mean-square fluctuations (RMSFs) of MDM2 were estimated using the coordinates of Cα atoms (Figure 5C). In Figure 5C, S1 represents loop L1, and S2 corresponds to the β-strands and loops between α2 and α1’, while S3 refers to β2’ and the loops between α1’ and α2’. It was observed that the S1, S2, and S3 regions of MDM2 exhibit high flexibility, particularly in the case of S2. The analysis highlights that the mobility of the S1, S2, and S3 domains is more pronounced in the 0Y7-MDM2 structure compared to the other structures. With reference to K23-MDM2, the binding of 0Y7 largely enhances the structural flexibility of the structural domains S1, S2, and S3, while the presence of the two peptide inhibitors, PDI6W and PDI, obviously weakens the structural flexibility of these two structural domains (Figure 5C,D), implying the different binding abilities of the four inhibitors to MDM2.

**Figure 6:**
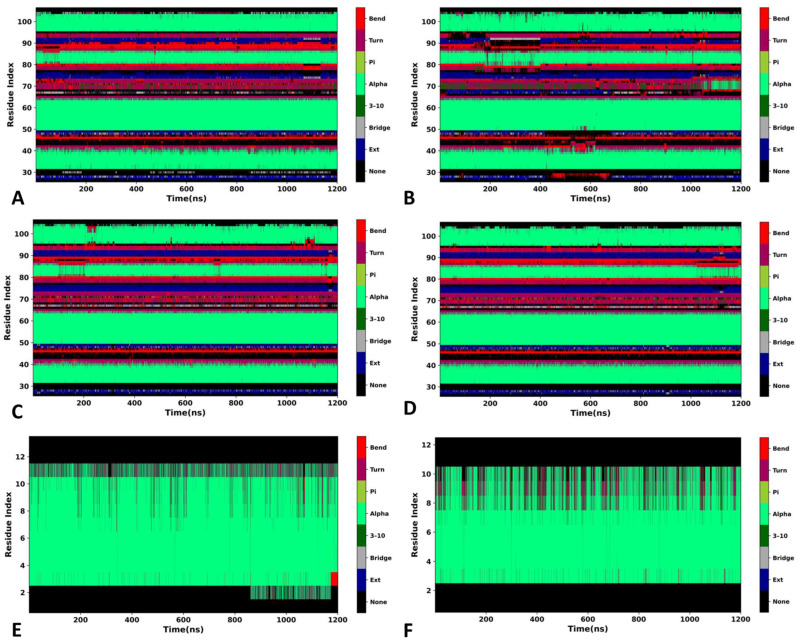
Stability of secondary structures of MDM2 and peptide inhibitors: (**A**) time evolution of secondary structure for MDM2 in the K23-MDM2 complex; (**B**) time evolution of secondary structure for MDM2 in the 0Y7-MDM2 complex; (**C**) time evolution of secondary structure for MDM2 in the PDI6W-MDM2 complex; (**D**) time evolution of secondary structure for MDM2 in the PDI-MDM2 complex; (**E**) time evolution of secondary structure for the peptide inhibitor PDI6W; (**F**) time evolution of secondary structure for the peptide inhibitor PDI.

To assess alterations in the secondary structures of MDM2 and the peptide inhibitors, a combination of the program CPPTRAJ and DSSP second-structure analysis [74] was used to investigate the changes in the secondary structures in three separate GaMD simulations. The time evolutions of the secondary structures for the K23-, 0Y7-, PDI6W- and PDI-bound MDM2 structures are displayed in Figure 6A–D, individually. The time evolutions of the secondary structures for the peptide inhibitors PDI6W and PDI are displayed in Figure 6E,F, individually. It is observed that the secondary structures of MDM2 and the peptide inhibitors hardly change throughout three separate GaMD simulations. The stability of these structures is favorable for the binding of inhibitors.

Based on the above analyses, the binding of peptide and non-peptide inhibitors yields different impacts on conformations of MDM2: (1) the binding of peptide inhibitors leads to more stable structures of MDM2 than non-peptide inhibitors; (2) the presence of peptide inhibitors induces more rigid structures in the S1, S2, and S3 domains. These results are in basic agreement with previous studies [35,36,37,38]. 

### 2.4. Comparative Calculations of Binding Free Energies

The binding free energies of K23, 0Y7, PDI6W, and PDI to MDM2 were assessed through MM-GBSA and SIE calculations so as to understand the binding preferences of the two types of inhibitors, and the results are shown in Table 1 and Table 2. In our calculations, 400 snapshots were extracted from the equilibrated cMD trajectories. It was found that the ranking of the calculated binding free energies was consistent with that determined by the known experimental data, implying the reliability of our free energy analyses [22,23,35,36,67,68].

As illustrated in Table 1, the binding free energies of K23, 0Y7, PDI, and PDI6W to MDM2 were −10.31, −12.61, −21.96, and −24.67 kcal·mol^−1^, respectively. Analysis of the energy components revealed that van der Waals energies ($∆E_{vdW}$) are the primary favorable contributors to inhibitor binding. Non-polar solvation energies ($∆G_{surf}$) also provide favorable contributions to the binding process. However, the contributions of entropy changes (−T∆S) to free energies greatly impair the binding of the four inhibitors to MDM2. Electrostatic terms ($∆E_{ele}$) also favor inhibitor binding, but polar solvation energies ($∆G_{gb}$) provide opposite contributions for the binding of inhibitors. On the whole, the sum of $∆E_{ele}$ and $∆G_{gb}$ in the current complexes is unfavorable for the binding of inhibitors. Therefore, two favorable forces, $∆E_{vdW}$ and $∆G_{surf}$, mostly drive the binding of the four inhibitors to MDM2, which agrees well with previous experimental analyses [35,36,37,38]. This result suggests that the optimization of van der Waals interactions and non-polar solvation energies may lead to the potent inhibition of MDM2.

According to Table 2, the calculated binding free energies for K23, 0Y7, PDI, and PDI6W to MDM2 using the SIE method are −7.19, −7.25, −10.53, and −10.79 kcal·mol^−1^, respectively. More importantly, the ranking of binding free energies predicted by the SIE method also agrees with that of the experimental values, which further verifies the reliability of our current results. The reaction energy ($∆G^{R}$) associated with the desolvation of polar groups always impedes inhibitor bindings (Table 2). As seen in Table 2, the unfavorable reaction energy of the polar groups is partially compensated by the favorable intermolecular Coulomb interaction ($∆E_{c}$). Additionally, intermolecular VDWIs ($∆E_{vdW}$) also provide partial compensation to this unfavorable effect.

Through the above results, the binding free energies predicted by the MM-GBSA and SIE methods show that the four current inhibitors yield tight associations with MDM2, implying that they have strong competitive ability in binding to MDM2 relative to p53. Furthermore, the VDWIs of the inhibitors with MDM2 play key roles in the binding of the two types of inhibitors, which implies that the hydrophobic groups of the peptide and non-peptide inhibitors, such as alkyls and aromatic rings, are significant molecular structures to be considered in future drug design in relation to p53-MDM2 interactions. Meanwhile, VDWIs should be paid more attention in the development of clinically available inhibitors targeting p53-MDM2 interactions.

### 2.5. Interaction Network of Inhibitors with MDM2

**Figure 7:**
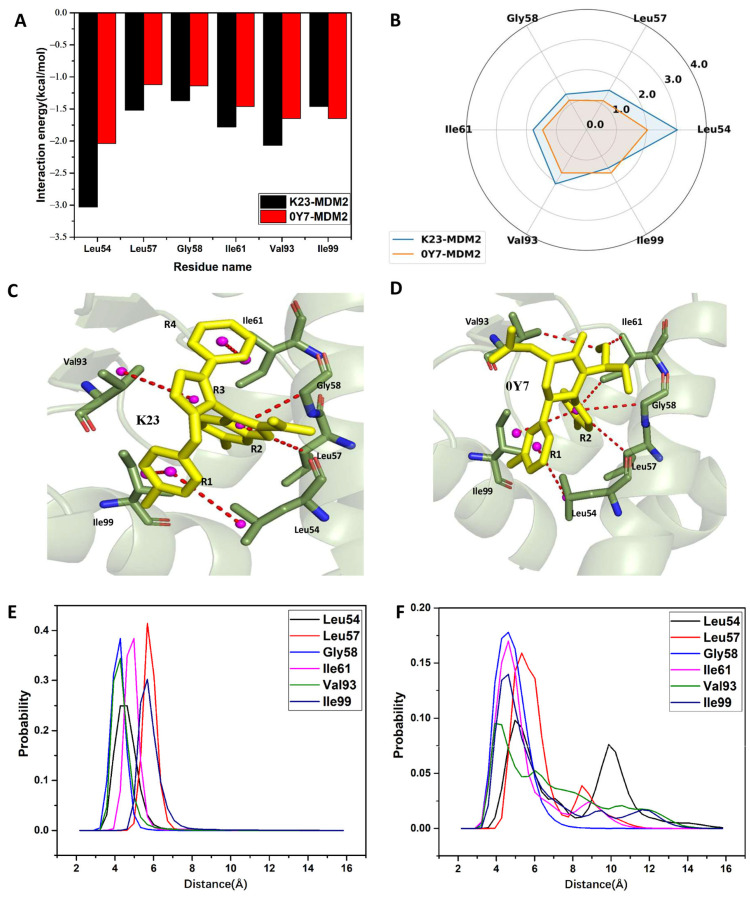
Interaction network of inhibitors K23 and 0Y7 with MDM2: (**A**) the key residues playing important roles in the binding of inhibitors to MDM2; (**B**) the radar representation of inhibitor–residue interactions in MDM2; (**C**) hydrophobic interactions of inhibitors with residues in the K23-MDM2 complex; (**D**) hydrophobic interactions of inhibitors with residues in the 0Y7-MDM2 complex; (**E**) the probability distribution of the distances between K23 and the key residues in MDM2; and (**F**) the probability distribution of the distances between 0Y7 and the key residues in MDM2.

**Figure 8:**
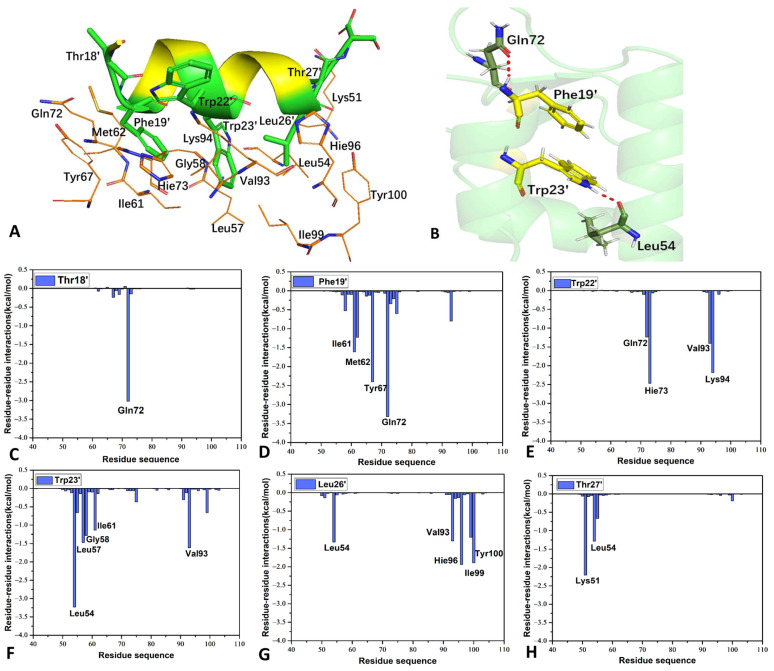
Interaction network of PDI6W inhibitor with MDM2: (**A**) relative geometric positions of the key residues in the pDl6W-MDM2 complex; (**B**) hydrogen bonding interactions in the pDl6W-MDM2 complex; and (**C**–**H**) residue–residue interaction spectrum between the pDl6W inhibitor and MDM2.

**Figure 9:**
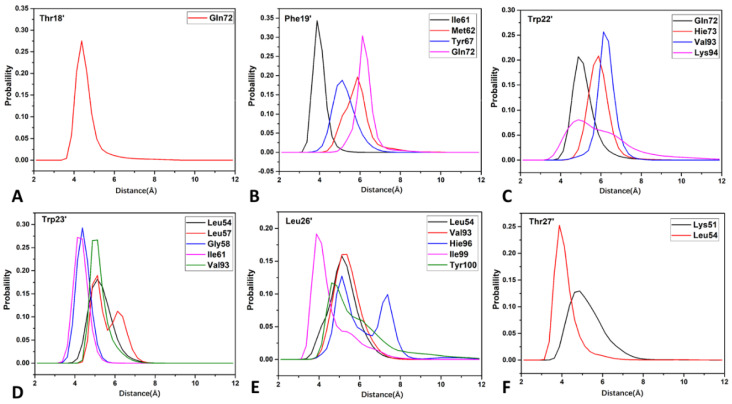
Probability distributions of the distances relating to key inhibitor–residue interactions for the PDI6W-MDM2 complex: (**A**) the distance between Thr18’ and the key residue in MDM2; (**B**) the distances between Phe19’ and the key residues in MDM2; (**C**) the distances between Trp22’ and the key residues in MDM2; (**D**) the distances between Trp23’ and the key residues in MDM2; (**E**) the distances between Leu26’ and the key residues in MDM2; and (**F**) the distances between Thr27’ and the key residues in MDM2.

To understand the contributions of individual residues to inhibitor–MDM2 binding, a residue-based free energy decomposition method was utilized to estimate inhibitor–residue interactions and residue–residue interactions (Figure 7, Figure 8 and Figures S2 and S3). The probability distributions of the distances associated with crucial inhibitor–residue interactions were analyzed (Figure 9 and Figure S4). Furthermore, the geometric aspects of hydrophobic interactions and hydrogen bonding interactions (HBIs) were visually illustrated for better comprehension (Figure 7C,D, Figure 8A,B and Figure S3A,B).

In the K23-MDM2 complex, K23 establishes interactions of strength exceeding 1 kcal/mol with six residues, specifically Leu54, Leu57, Gly58, Ile61, Val93, and Ile99 (Figure 7A,B and Figure S2A). As illustrated in Figure 7C, the alkyl groups of residues Leu54 and Ile99 are close to the hydrophobic ring R1 of K23, leading to the formation of CH-π interactions between them. The respective interaction energies for these interactions are −3.03 and −1.46 kcal/mol for Leu54 and Ile99 (Figure 7A). The distances for the mass centers of the sidechains of Leu54 and Ile99 away from the mass center of the ring R1 are, respectively, distributed at 4.63 and 5.68 Å (Figure 7E), which verifies the hydrophobic interactions of these two residues with K23. The alkyl groups of Leu57 and the CH group of Gly58 form multiple CH-π contacts with the R2 ring of K23 (Figure 7C), leading to CH-π interaction energies of −1.52 and −1.37 kcal/mol for residues Leu57 and Gly58, respectively (Figure 7A,B). The distances for the carbon atoms of the alkyl groups of Leu57 and Gly58 from the mass center of the ring R2 are distributed at 5.68 and 4.28 Å (Figure 7E), respectively, which verifies the existence of CH-π interactions. The alkyl group of Val93 engages in the CH-π contacts with the hydrophobic ring R3 of K23, while the alkyl moiety of Ile61 forms CH-π contacts with the hydrophobic group R4 of K23 (Figure 7C). As a result, Val93 and Ile61 separately provide the interaction energies of −2.07 and −1.78 kcal/mol for the K23-MDM2 binding (Figure 7A,B). The distance between the center of mass of the alkyl group from Val93 and that of ring R3 is situated at 4.28 Å (Figure 7E), indicating the presence of a CH-π interaction between K23 and Val93. Similarly, the distance between the center of mass of the alkyl group from Ile61 and that of ring R4 is 4.98 Å (Figure 7E), demonstrating the existence of the CH-π interaction between K23 and Ile61. Moreover, K23 establishes a hydrogen bonding interaction with Leu54 with an occupancy exceeding 98.58%, suggesting the stability of this hydrogen bond throughout the GaMD simulations (Table 3). These analyses align with previous statistical examinations and other research findings [35,36,37,38]. 

In the 0Y7-MDM2 complex, 0Y7 produces interactions stronger than 1.0 kcal/mol with six residues, including Leu54, Leu57, Gly58, Ile61, Val93, and Ile99 (Figure 7A and Figure S2B). The alkyl groups of Leu57, Gly58, ILE61, and ILE99 establish several CH-π contacts with the hydrophobic ring R2 of 0Y7, resulting in interaction energies of −1.12, −1.14, −1.46, and −1.65 kcal/mol for Leu57, Gly58, ILE61, and ILE99, respectively (Figure 7A,B,D). The distances of the mass centers for the alkyl groups of Leu57, Gly58, ILE61, and ILE99 away from the ring R2 are situated at 5.33, 4.63, 4.63, and 4.63 Å, individually, as depicted in Figure 7F. The alkyl group of Leu54 forms CH-π contacts with the ring R1 of 0Y7, and its corresponding interaction energy is −2.04 kcal/mol (Figure 7A,B). The distance between the center of mass of the alkyl group from Leu54 and that of ring R1 is 4.98 Å (Figure 7F). The alkyl of Val93 structurally forms a CH–CH interaction with that of 0Y7, which provides an energy contribution of −1.65 kcal/mol to the 0Y7-MDM2 binding (Figure 7A,D). Moreover, the distance of 3.93 Å between the carbon atom of the alkyl group from Val93 and that of 0Y7 verifies the existence of the CH-CH interaction (Figure 7F).

PDI6W-MDM2 interactions are illustrated with the relative positions between key residues from PDl6W and MDM2 (Figure 8). Seven residues of PDl6W can strongly interact with MDM2. The Thr18’ of the PDl6W inhibitor engages in a significant CH-CH interaction of −3.02 kcal/mol with Gln72 of MDM2 (Figure 8A,C), while the distance between the center of mass of the alkyl group from Gln72 and that of Thr18’ is 4.34 Å (Figure 9A). The Phe19’ of the PDl6W inhibitor exhibits strong interactions with four MDM2 residues, including Ile61, Met62, Tyr67, and Gln72. Specifically, the interaction energy between Phe19’ and Tyr67 is −2.40 kcal/mol, aligning with the π-π interaction between the benzene rings of these two residues in the spatial structure (Figure 8A,D). The interaction energies between Phe19’ and residues Ile61, Met62, and Gln72 are −1.61, −1.23, and −3.32 kcal/mol, respectively (Figure 8D). These energy contributions primarily arise from the CH-π interactions between the CH groups of residues Ile61, Met62, and Gln72 and the benzene ring of Phe19’ (Figure 8A). As illustrated in Figure 9B, the distances between the mass centers of the alkyl groups of Ile61, Met62, and Tyr67 and that of Phe19’ are 3.88, 5.88, and 5.13Å, respectively, which demonstrates the existence of the aforementioned CH-π interactions. A hydrogen bond with an occupancy of 76.31% appears between Phe19’ and Gln72 (Table 3 and Figure 8B), implying that this hydrogen bond is stable. Structurally, the CH groups of Gln72, Val93, and Lys94 engage in the CH-π interactions with Trp22’ of the pDl6W inhibitor (Figure 8A), resulting in interaction energies of −1.24, −1.41, and −2.18 kcal/mol, respectively (Figure 8E). His73 establishes a π-π interaction of −2.47 kcal/mol with Trp22’ (Figure 8A,E). The distances for the mass centers of the alkyl groups of Gln72, His73, Val93, and Lys94 from those of Trp22’ are separately distributed at 4.88, 5.88, 6.13, and 4.88 Å (Figure 9C). The most robust interaction is observed between Trp23’ and Leu54, with an interaction energy of −3.23 kcal/mol (Figure 8F). This energy primarily originates from two sources: (1) the alkyl group of Leu54 forms a CH-π interaction with Trp23’ (Figure 8A), and (2) a hydrogen bond with a 97.62% occupancy is formed between Leu54 and Trp23’ (Table 3 and Figure 8B). The interaction energies between Trp23’ and Leu57, Gly58, Ile61, and Val93 are −1.47, −1.28, −1.14, and −1.61 kcal/mol, respectively (Figure 8F), reflecting the structural agreement of the CH-π interactions between the CH groups of these four residues and the hydrophobic ring of Trp23’ (Figure 8A). The distances between the mass centers of the alkyl groups of Leu54, Leu57, Gly58, Ile61, and Val93 and those of Trp23’ are populated at 5.13, 5.13, 4.63, 4.38, and 5.13 Å (Figure 9D), which supports the abovementioned CH-π interactions involving Trp23’. The Leu26’ residue of the PDl6W inhibitor exhibits significant interactions with five MDM2 residues, including Leu54, Val93, His96, Ile99, and Tyr100. The interaction energies between Leu26’ and Leu54, Val93, and Ile99 are −1.33, −1.30, and −1.20 kcal/mol, respectively (Figure 8G), and are predominantly driven by the CH-CH interaction between the alkyl groups of Leu54, Val93, and Ile99 and the alkyl group of Leu26’ (Figure 8A). Additionally, the interaction energies between Leu26’ and His96 and Tyr100 are −1.94 and −1.89 kcal/mol (Figure 8G), which is consistent with the CH-π interaction of His96 and Tyr100 with the alkyl groups of Leu26’ (Figure 8A). In Figure 9E, the distances between the mass centers of the hydrophobic groups of Leu54, Val93, His96, Ile99, and Tyr100 and those of Trp23’ are located at 5.13, 5.38, 5.13, 3.88, and 4.63 Å, respectively, which further reveals key residues interacting with Trp23’. The interaction energies of Thr27’ in PDI6W with residues Lys51 and Leu54 are −2.21 and −1.28 kcal/mol, coming structurally from the CH-CH interactions (Figure 8A,H). The distances between the mass centers of the alkyl groups of Lys51 and Leu54 from those of Trp26’ are 4.88 and 3.88Å, as illustrated in Figure 9F, implying the existence of these key interactions.

As seen in Figures S3 and S4, the PDI inhibitor binds to MDM2 in a mode similar to pDl6W. A hydrogen bond with an occupancy of 81.36% appears between Phe19’ and Gln72 (Table 3 and Figure S3B). Additionally, Leu54 forms a hydrogen bond with Trp23’, and its occupancy is 95.73% (Table 3 and Figure S3B). Compared to the residue–residue interaction in PDI6W, the interaction energy between Tyr22’ and Lys94 is lower than 1.0 kcal/mol, which indicates that there is a weak CH-π contact between the alkyl moiety of Lys94 and Tyr22’ (Figure S3E). The probability distributions of the distances relating to key inhibitor–residue interactions for PDI-MDM2 are illustrated in Figure S4. The work by Liu et al. showed that D-peptide inhibitors can form favorable interactions with residues Leu54, Leu57, Ile61, Tyr67, Val93, His96, and Tyr100, which agrees well with our current calculated results [75,76]. The study of Strizhak et al. revealed that a stapled peptide produces interactions with Leu54, Ile61, Tyr67, Gln72, Val93, His96, and Ile99, supporting our current findings [77].

In previous experimental and theoretical works [14], p53 has yielded strong interactions with Leu54, Leu57, Gly58, Ile61, Val93, Ile99, etc., which not only supports our current results well, but also implies that four inhibitors occupy the binding sites of p53 in MDM2, impairing the binding of p53. Moreover, the sidechains of key residues revealed by our calculations play vital roles in the binding of inhibitors to MDM2. More importantly, the CH-π, CH-CH, and π-π interactions between individual residues of MDM2 and the inhibitors drive the binding of K23, 0Y7, PDl6W, and PDI to MDM2, which should be paid special attention in future drug design in relation to p53-MDM2 interactions. Therefore, it is of high significance to rationally optimize the interactions of inhibitors with the sidechains of key residues in MDM2 for the design of efficient inhibitors.

## 3. Materials and Methods

### 3.1. System Preparation

The initial atomic coordinates of the K23-, 0Y7-, PDI6W-, and PDI-MDM2 complexes were taken from the Protein Data Bank (PDB), corresponding to PDB entries 3LBK, 4HBM, 3JZR, and 3G03, respectively. As there are differences in the residue sequences of MDM2, residues of Thr26-Arg105 in MDM2 were utilized to construct our simulation systems. The residues from the two peptide inhibitors, PDI6W and PDI, were canonical amino acids. The protonation states of the MDM2 residues were validated using the H++ 3.0 program [78]. Rational protonation states were assigned to each MDM2 residue, and any missing hydrogen atoms in the crystal structures were added using the Leap module in Amber20 [79,80]. The structures of two inhibitors, K23 and 0Y7, were optimized at a semi-empirical AM1 level, and subsequently, BCC charges [81] were assigned to each atom of the two inhibitors using the Antechamber module in Amber20 [82]. The *ff*19SB force field [83] was employed to parameterize MDM2, while the general Amber force field (GAFF2) [84,85] was used to derive force-field parameters for K23 and 0Y7. The systems, comprising K23-MDM2, 0Y7-MDM2, PDI6W-MDM2, and PDI-MDM2, were solvated in an octahedral periodic water box with a 10.0 Å buffer to mimic a solvent environment. The force-field parameters for the water molecules were based on the TIP3P model [86,87]. To ensure neutral simulation systems, an appropriate number of sodium ions (Na^+^) and chloride ions (Cl^−^) at a concentration of 0.15 M NaCl were added to the water box. The parameters for the Na^+^ and Cl^−^ ions were adopted from the work of Joung et al. [88,89].

### 3.2. Multiple Independent Gaussian-Accelerated Molecular Dynamics

To address potential bad contacts between atoms arising from the initialization of the four current MDM2-related systems, each system underwent a two-step minimization process. This included a 5000-cycle steepest descent minimization followed by a 10,000-cycle conjugate gradient minimization. The optimized systems were gradually heated from 0 to 300 K over 1 ns in the canonical ensemble (NVT), utilizing a weak harmonic restraint of 2 kcal·mol^−1^‧Å^2^ on heavy atoms. Subsequently, the four systems were further equilibrated at 300 K under an isothermal−isobaric ensemble (NPT). A 2 ns NPT simulation was then conducted to maintain the system density at 1.01 g/cm^3^. Finally, three 2.4 ns independent cMD simulations were separately performed on the four systems during the NVT with periodic boundary conditions using the particle mesh Ewald method (PME). In each independent cMD simulation, the initial atomic velocities were randomly assigned with the Maxwell distribution.

The well-equilibrated systems served as the starting points for three independent Gaussian-accelerated molecular dynamics (GaMD) simulations. GaMD simulations employ a harmonic boost potential to reduce free energy barriers in biomolecules and enhance the conformational sampling of systems. In GaMD simulations, if the potential energy $V(\overset{⃑}{r})$ of the system is lower than a threshold energy E, $V(\overset{⃑}{r})$ is revised to $V^{*}(\overset{⃑}{r})$, according to Equations (1) and (2) below:

(1) $$V^{*}\left(\overset{⃑}{r}\right)=V\left(\overset{⃑}{r}\right)+∆V(\overset{⃑}{r})$$

(2) $$∆V\left(\overset{⃑}{r}\right)=\left\{\begin{array}{c} 0,\;\;\;\;\;\;\;\;\;\;\;\;\;\;\;\;\;\;\;\;\;\;\;\;\;\;\;\;V(\overset{⃑}{r})\geq E\\ \frac{1}{2}k{\left(E-V\left(\overset{⃑}{r}\right)\right)}^{2},\;\;\;V\left(\overset{⃑}{r}\right)<E \end{array}\right.$$

In the above equations, the parameter k represents the harmonic force constant. The parameters E and k can be adjusted according to the enhanced sampling principles defined in Equations (3) and (4), as shown below:

(3) $$V_{max}\leq E\leq V_{min}+\frac{1}{k}$$

(4) $$k=k_{0}\frac{1}{V_{max-}V_{min}}$$

If E is designated as the lower bound ($E=V_{max}$), then $k_{0}$ can be determined using Equation (5):

(5) $$k_{0}=min(1.0,\;\frac{\sigma_{0}}{\sigma_{V}}\cdot \frac{V_{max}-V_{min}}{V_{max}-V_{avg}})$$

On the contrary, if E is set as the upper bound ($E=V_{min}+\frac{1}{k}$), then $k_{0}$ is derived from Equation (6):

(6) $$k_{0}=(1.0-\frac{\sigma_{0}}{\sigma_{V}})\cdot (\frac{V_{max}-V_{min}}{V_{avg}-V_{min}})$$

where the three energy parameters $V_{max}$, $V_{min}$, and $V_{avg}$ indicate the maximum, minimum, and averaged potential energies of the simulated systems extracted from the previous cMD simulations, respectively. The parameter $\sigma_{V}$ represents the standard deviation of the system’s potential energies, and $\sigma_{0}$ is a user-defined upper limit for accurate reweighting. In our current study, 1.2 μs GaMD simulations, composed of three independent simulations of 400 ns, were separately performed on four current MDM2-related systems. To facilitate deep learning (DL) and post-processing analyses, three independent GaMD trajectories were combined into a single GaMD trajectory (SGT), and the CPPTRAJ module integrated with Amber was used to extract data for insights into the function of MDM2-related systems. A program called PyReweighting, developed by Miao et al. [90], was utilized to accurately reweight and identify the original free energy profiles of our current systems. 

All cMD and GaMD simulations employed the SHAKE algorithm to constrain the chemical bonds between the hydrogen atoms and heavy atoms [91]. The Langevin thermostat, bringing a collision frequency of 2.0 ps^−1^, was utilized to tune the temperatures of the four MDM2-related systems [92]. Non-bonded interactions were estimated using the particle mesh Ewald (PME) method [93] with a 12 Å cutoff. The simulations were executed using the pmemd.cuda program implemented in Amber20 [94,95]. 

### 3.3. Deep Learning

To investigate the impacts of inhibitor binding on the internal structures of MDM2, DL was utilized to identify differences in residue contacts. The residue contact maps for each snapshot of MDM2 were computed using the Python packages MDTraj and Contact Map Explorer [96]. Contact was defined as ≤4.5 Å between any Cα atoms of two proteins. The resulting 80 × 80 residue contacts were converted into grayscale images of 80 × 80 pixels for analysis by a two-dimensional (2D) convolutional neural network (CNN). A total of 160,000 images were generated for each MDM2-related system, with 80% randomly selected for training and the remaining 20% used for validation. The 2D-CNN model was constructed using the PyTorch package, consisting of three convolutional layers with a 1 × 1 kernel size and 16, 32, and 32 filters, followed by three fully connected layers. The first two fully connected layers comprised 512 and 128 filters with a dropout rate of 0.5 each, while the final fully connected layer served as the classification layer for inhibitor-bound MDM2.

Throughout the 2D-CNN architecture, the “ReLu” activation function was employed in all layers, with the “softmax” activation function used at the classification layer. A maximum pooling layer with a 2 × 2 kernel size was added after each convolutional layer. Backpropagation via vanilla gradient-based pixel attribution [97] was utilized to estimate an attention map of the residue contact gradients to aid in discriminating the functional differences in MDM2 induced by inhibitor binding. The residue contact map was represented using the most populated structural cluster of each MDM2-related system. Our DL program was rewritten using PyTorch based on the work of Miao’s group [59].

### 3.4. Principal Component Analysis and Dynamic Cross-Correlation Maps

PCA is a crucial technique for deciphering conformational changes in proteins. In this research, PCA was conducted by diagonalizing the covariance matrix C constructed from the Cα atom coordinates of MDM2, as outlined in Equation (7):

(7) $$C=<(q_{i}-<q_{i}>){\left(q_{j}-<q_{j}>\right)}^{T}>$$

where $q_{i}$ and $q_{j}$ represent the Cartesian coordinates of the *i*th and *j*th Cα atoms from MDM2, while <$q_{i}$> and <$q_{j}$> denote their average positions across conformational ensembles obtained from MIGaMD simulations. The eigenvector and the eigenvalue generated by the diagonalization of the covariance matrix characterize the concerted movement of the structural domains and the fluctuation amplitude along a given eigenvector, respectively. For this study, PCA was conducted using the CPPTRAJ program [98] in the Amber suite.

### 3.5. Construction of Free Energy Landscapes

To investigate the influences of peptide and non-peptide inhibitors on the free energy profiles of MDM2, projections of GaMD trajectories onto the first two eigenvectors served as RCs for constructing free energy landscapes (FELs). During the reweighting process in GaMD simulations, reweighted free energy $F\left(A\right)=-k_{B}Tln(\rho_{A})$ is calculated as

(8) $$F\left(A\right)=F^{*}\left(A\right)-\sum_{k=1}^{2}\frac{\beta^{k}}{k!}C_{k}+F_{C}$$

where $F^{*}\left(A\right)=-k_{B}Tlnp^{*}\left(A\right)$ represents the modified free energy obtained from the GaMD simulations, $F_{C}$ denotes a constant, and ${\beta =k}_{B}T$. The probability distribution $p^{*}\left(A\right)$ of the selected RCs from the GaMD simulations can be reweighted to match the canonical ensemble distribution $\rho_{A}$. All free energy reweighting calculations were performed using the PyReweighting program developed by Miao et al. A detailed description for the reweighting is given in the work of Miao et al. [90].

### 3.6. Binding Free Energies

To evaluate the binding of the two types of inhibitors to MDM2, MM-GBSA and SIE methods were adopted to calculate binding free energies. In the MM-GBSA method, enthalpy changes (∆H) and entropy changes (-T∆S) play essential roles in ligand associations. The binding free energies of the non-peptide inhibitors K23 and 0Y7 and the peptide inhibitors PDI6W and PDI to MDM2 were calculated using the MM-GBSA method based on the following Equation (9):

(9) ∆G=∆H−T∆S

where ∆H is calculated using Equation (10):

(10) $$∆H=∆E_{ele}+∆E_{vdW}+∆G_{gb}+∆G_{surf}$$

where electrostatic interactions (EIs, $∆E_{ele}$) and van der Waals interactions (VDWIs, $∆E_{vdW}$) can be estimated using molecular mechanics and the *ff*19SB force field. Polar solvation free energies (PSFEs, $∆G_{gb}$) were estimated using the generalized Born (GB) model proposed by Onufriev et al. [99]. Non-polar solvation free energies (NPSFEs, $∆G_{surf}$) were calculated based on the following empirical Equation (11):

(11) $$∆G_{surf}=\gamma \times ∆SASA+\beta$$

where the term ∆SASA represents the variation in the solvent-accessible surface area (SASA) mediated by the binding of inhibitors. Entropy changes (-T∆S) were computed using the MMPBSA.py program within the Amber20 software [100]. The two parameters γ and β were set as 0.0072 kcal·mol·Å^−2^ and 0.0 kcal·mol^−1^, respectively [101].

In the SIE method, the SIE function [66] to calculate inhibitor–protein binding free energies is expressed as follows in Equation (12):

(12) $$∆G_{bind}(\rho ,D_{in},\alpha ,\gamma ,C)=\alpha \times [E_{c}(D_{in})+∆G^{R}+E_{vdW}+\gamma \cdot ∆MSA(\rho )]+C$$

where $E_{c}$ and $E_{vdW}$ represent the intermolecular Coulomb and van der Waals interaction energies in the bound state, respectively. $∆G^{R}$ signifies the change in the reaction field energy caused by the binding of an inhibitor, which was determined by solving the Poisson equation using the boundary element method (BRI BEM) [102,103] and a solvent probe with a variable radius of 1.4 Å [104]. γ·∆MSA corresponds to the change in the molecular surface area upon binding. The parameters ρ, $D_{in}$, γ, and *C* are the Amber van der Waals radii linear scaling coefficient, the solute interior dielectric constant, the molecular surface area coefficient, and a constant, respectively. The parameter α relates to the overall proportionality coefficient associated with the loss of conformational entropy upon binding [105]. The optimized values of these parameters are as follows: α=0.1048, $D_{in}=2.25$, ρ=1.1, γ=0.0129kcal/(mol·Å), and $C=-2.89kcal\cdot {mol}^{-1}$ [66,106]. The SIE calculations were performed with the program Sietraj [106].

## 4. Conclusions

Investigating the molecular mechanism inhibiting the MDM2-p53 interaction is pivotal for a deeper understanding of the treatment approaches used for cancers. To enhance the conformation sampling of the inhibitor–MDM2 complexes, three individual GaMD simulations of 1.2 μs, each running for 400 ns, were conducted on four MDM2-related systems. Through GaMD trajectory-based deep learning, key functional domains of MDM2, predominantly situated at helices α2 and α2’, as well as the β-strands and loops between α2 and α2’, were identified. The calculated binding free energies obtained through the MM-GBSA and SIE methods not only highlight that VDWIs are the primary driving forces in the binding of inhibitors to MDM2, but also indicate that peptide inhibitors establish more interaction contacts with MDM2 than non-peptide inhibitors. The results of the PCA and FELs suggest that the piperidinone inhibitor exerts a significant influence on free energy profiles, exhibiting higher fluctuation amplitudes along primary eigenvectors. The analysis of the interaction network reveals crucial residues involved in inhibitor binding and identifies potential targeting sites for drug design in relation to MDM2. This work is anticipated to contribute significant theoretical aid to the development of potential inhibitors for inhibiting the p53-MDM2 interaction.

## Figures and Tables

**Table 1:** Binding free energies of inhibitors to MDM2 obtained by MM-GBSA method.

Complex	K23-MDM2		0Y7-MDM2		PDI-MDM2		PDI6W-MDM2	
	Average	Std	Average	Std	Average	Std	Average	Std
$∆E_{ele}$	−105.32	9.38	−2.43	7.63	−222.94	36.30	−250.13	33.93
$∆E_{vdW}$	−37.68	3.46	−34.25	4.05	−61.75	4.29	−63.96	4.94
$∆G_{gb}$	119.19	9.40	12.76	6.45	244.68	34.79	272.15	32.41
$∆G_{surf}$	−4.63	0.38	−4.48	0.58	−7.80	0.57	−8.10	0.60
∆Ggbele a	13.87	1.67	10.33	2.96	21.73	3.55	22.03	4.00
−T∆S	−18.12	4.63	−15.80	5.37	−25.86	6.28	−25.38	6.14
∆Gbind b	−10.31		−12.61		−21.96		−24.67	
∆Gexp c	−7.89		−9.98		−10.01		−10.18	

Note: Standard errors are given in parentheses. ∆aGgbele=∆Eele+∆Ggb. ∆Gbind=∆Eele+∆Ggb+∆Evdw+∆Gsurf−T∆Sb. ∆Gexpa: The experimental values were derived from the experimental Ki values in reference [22,67,68] using the equation ${∆G}_{exp}=-RTln{IC}_{50}$.

**Table 2:** Binding free energies of MDM2 to inhibitors calculated by SIE method.

Complex	K23-MDM2		0Y7-MDM2		PDI-MDM2		PDI6W-MDM2	
	Average	Std	Average	Std	Average	Std	Average	Std
$∆E_{vdW}$	−36.07	2.97	−36.96	3.79	−62.01	4.21	−63.96	4.94
$∆E_{c}$	−47.60	4.02	−3.06	4.14	−99.20	17.98	−111.21	15.07
γ·∆MSA	−6.59	0.30	−7.14	0.48	−10.85	0.65	−11.33	0.63
$∆G^{R}$	49.23	4.16	5.56	2.71	99.09	16.43	111.10	13.82
∆Gbind	−7.19		−7.25		−10.53		−10.79	
∆Gexp a	−7.89		−9.98		−10.01		−10.18	

Note: Standard errors are given in parentheses. ∆Gexpa: The experimental values were derived from the experimental Ki values in reference [22,67,68] using the equation ${∆G}_{exp}=-RTln{IC}_{50}$.

**Table 3:** The hydrogen bonds formed between key residues and inhibitors of MDM2.

Inhibitor	Donor	Acceptor	^a^ Distance(Å)	^a^ Angle(°)	^b^ Occupied(%)
K23	K23:N8-H4	Leu54:O	2.89	157.08	98.58
PDI6W	Phe19’: N-H	Gln72: OE1	2.98	154.03	76.31
	Trp23’: NE1- HE1	Leu54: O	2.89	148.67	97.62
PDI	Phe19’: N-H	Gln72: OE1	2.98	154.83	81.36
	Trp23’: NE1- HE1	Leu54: O	2.92	149.58	95.73

^a^ The hydrogen bonds are determined by a donor–acceptor atom distance of <3.5 Å and acceptor–H–donor angle of >120°. ^b^ Occupancy is used to evaluate the stability and strength of the hydrogen bond.

## Data Availability

Data are contained within the article and Supplementary Materials.

## Supplementary Materials

The following supporting information can be downloaded at: https://www.mdpi.com/article/10.3390/molecules29143377/s1, Figure S1: Eigenvalues and their corresponding proportions; Figure S2: Interactions of inhibitors with separate residues in K23-MDM2 and 0Y7-MDM2; Figure S3: Interaction network of PDI inhibitor with MDM2; Figure S4: Probability distributions of the distances relating to key inhibitor–residue interactions for PDI-MDM2.

## References

[1] Patil M.R., Bihari A. (2022). A comprehensive study of p53 protein. J. Cell. Biochem..

[2] Ozaki T., Nakagawara A. (2011). p53: The attractive tumor suppressor in the cancer research field. J. Biomed. Biotechnol..

[3] Yoshida K., Miki Y. (2010). The cell death machinery governed by the p53 tumor suppressor in response to DNA damage. Cancer Sci..

[4] Bykov V.J.N., Eriksson S.E., Bianchi J., Wiman K.G. (2018). Targeting mutant p53 for efficient cancer therapy. Nat. Rev. Cancer.

[5] Khoo K.H., Verma C.S., Lane D.P. (2014). Drugging the p53 pathway: Understanding the route to clinical efficacy. Nat. Rev. Drug Discov..

[6] Wang W., Albadari N., Du Y., Fowler J.F., Sang H.T., Xian W., McKeon F., Li W., Zhou J., Zhang R. (2024). MDM2 Inhibitors for Cancer Therapy: The Past, Present, and Future. Pharmacol. Rev..

[7] Zafar A., Khan M.J., Naeem A. (2023). MDM2- an indispensable player in tumorigenesis. Mol. Biol. Rep..

[8] Wade M., Li Y.C., Wahl G.M. (2013). MDM2, MDMX and p53 in oncogenesis and cancer therapy. Nat. Rev. Cancer.

[9] Mendoza M., Mandani G., Momand J. (2014). The MDM2 gene family. Biomol. Concepts.

[10] Momand J., Villegas A., Belyi V.A. (2011). The evolution of MDM2 family genes. Gene.

[11] Tan B.X., Liew H.P., Chua J.S., Ghadessy F.J., Tan Y.S., Lane D.P., Coffill C.R. (2017). Anatomy of Mdm2 and Mdm4 in evolution. J. Mol. Cell Biol..

[12] Yu D.H., Xu Z.Y., Mo S., Yuan L., Cheng X.D., Qin J.J. (2020). Targeting MDMX for Cancer Therapy: Rationale, Strategies, and Challenges. Front. Oncol..

[13] Sanford J.D., Yang J., Han J., Tollini L.A., Jin A., Zhang Y. (2021). MDMX is essential for the regulation of p53 protein levels in the absence of a functional MDM2 C-terminal tail. BMC Mol. Cell Biol..

[14] Kussie P.H., Gorina S., Marechal V., Elenbaas B., Moreau J., Levine A.J., Pavletich N.P. (1996). Structure of the MDM2 oncoprotein bound to the p53 tumor suppressor transactivation domain. Science.

[15] Koo N., Sharma A.K., Narayan S. (2022). Therapeutics Targeting p53-MDM2 Interaction to Induce Cancer Cell Death. Int. J. Mol. Sci..

[16] Nayak S.K., Khatik G.L., Narang R., Monga V., Chopra H.K. (2018). p53-Mdm2 Interaction Inhibitors as Novel Nongenotoxic Anticancer Agents. Curr. Cancer Drug Targets.

[17] Rasafar N., Barzegar A., Mehdizadeh Aghdam E. (2020). Design and development of high affinity dual anticancer peptide-inhibitors against p53-MDM2/X interaction. Life Sci..

[18] Lemos A., Leão M., Soares J., Palmeira A., Pinto M., Saraiva L., Sousa M.E. (2016). Medicinal Chemistry Strategies to Disrupt the p53-MDM2/MDMX Interaction. Med. Res. Rev..

[19] Chen S., Li X., Li Y., Yuan X., Geng C., Gao S., Li J., Ma B., Wang Z., Lu W. (2022). Design of stapled peptide-based PROTACs for MDM2/MDMX atypical degradation and tumor suppression. Theranostics.

[20] Rasafar N., Barzegar A., Mehdizadeh Aghdam E. (2020). Structure-based designing efficient peptides based on p53 binding site residues to disrupt p53-MDM2/X interaction. Sci. Rep..

[21] Wang Y.T., Cheng T.L. (2021). Computational modeling of cyclic peptide inhibitor-MDM2/MDMX binding through global docking and Gaussian accelerated molecular dynamics simulations. J. Biomol. Struct. Dyn..

[22] Phan J., Li Z., Kasprzak A., Li B., Sebti S., Guida W., Schönbrunn E., Chen J. (2010). Structure-based design of high affinity peptides inhibiting the interaction of p53 with MDM2 and MDMX. J. Biol. Chem..

[23] Czarna A., Popowicz G.M., Pecak A., Wolf S., Dubin G., Holak T.A. (2009). High affinity interaction of the p53 peptide-analogue with human Mdm2 and Mdmx. Cell Cycle.

[24] Fang Y., Liao G., Yu B. (2020). Small-molecule MDM2/X inhibitors and PROTAC degraders for cancer therapy: Advances and perspectives. Acta Pharm. Sin. B.

[25] Wang S., Chen F.E. (2022). Small-molecule MDM2 inhibitors in clinical trials for cancer therapy. Eur. J. Med. Chem..

[26] Beloglazkina A., Zyk N., Majouga A., Beloglazkina E. (2020). Recent small-molecule inhibitors of the p53-MDM2 protein-protein Interaction. Molecules.

[27] Rew Y., Sun D. (2014). Discovery of a small molecule MDM2 inhibitor (AMG 232) for treating cancer. J. Med. Chem..

[28] Liao G., Yang D., Ma L., Li W., Hu L., Zeng L., Wu P., Duan L., Liu Z. (2018). The development of piperidinones as potent MDM2-P53 protein-protein interaction inhibitors for cancer therapy. Eur. J. Med. Chem..

[29] Yu M., Wang Y., Zhu J., Bartberger M.D., Canon J., Chen A., Chow D., Eksterowicz J., Fox B., Fu J. (2014). Discovery of Potent and Simplified Piperidinone-Based Inhibitors of the MDM2-p53 Interaction. ACS Med. Chem. Lett..

[30] Gessier F., Kallen J., Jacoby E., Chène P., Stachyra-Valat T., Ruetz S., Jeay S., Holzer P., Masuya K., Furet P. (2015). Discovery of dihydroisoquinolinone derivatives as novel inhibitors of the p53-MDM2 interaction with a distinct binding mode. Bioorg. Med. Chem. Lett..

[31] Holzer P., Masuya K., Furet P., Kallen J., Valat-Stachyra T., Ferretti S., Berghausen J., Bouisset-Leonard M., Buschmann N., Pissot-Soldermann C. (2015). Discovery of a Dihydroisoquinolinone Derivative (NVP-CGM097): A Highly Potent and Selective MDM2 Inhibitor Undergoing Phase 1 Clinical Trials in p53wt Tumors. J. Med. Chem..

[32] de Weger V.A., de Jonge M., Langenberg M.H.G., Schellens J.H.M., Lolkema M., Varga A., Demers B., Thomas K., Hsu K., Tuffal G. (2019). A phase I study of the HDM2 antagonist SAR405838 combined with the MEK inhibitor pimasertib in patients with advanced solid tumours. Br. J. Cancer.

[33] Vu B., Wovkulich P., Pizzolato G., Lovey A., Ding Q., Jiang N., Liu J.J., Zhao C., Glenn K., Wen Y. (2013). Discovery of RG7112: A Small-Molecule MDM2 Inhibitor in Clinical Development. ACS Med. Chem. Lett..

[34] Kang M.H., Reynolds C.P., Kolb E.A., Gorlick R., Carol H., Lock R., Keir S.T., Maris J.M., Wu J., Lyalin D. (2016). Initial Testing (Stage 1) of MK-8242-A Novel MDM2 Inhibitor-by the Pediatric Preclinical Testing Program. Pediatr. Blood Cancer.

[35] Chen J., Zhang D., Zhang Y., Li G. (2012). Computational studies of difference in binding modes of peptide and non-peptide inhibitors to MDM2/MDMX based on molecular dynamics simulations. Int. J. Mol. Sci..

[36] Chen J., Wang J., Xu B., Zhu W., Li G. (2011). Insight into mechanism of small molecule inhibitors of the MDM2-p53 interaction: Molecular dynamics simulation and free energy analysis. J. Mol. Graph. Model..

[37] Niu R.J., Zheng Q.C., Zhang J.L., Zhang H.X. (2013). Molecular dynamics simulations studies and free energy analysis on inhibitors of MDM2-p53 interaction. J. Mol. Graph. Model..

[38] Hu G., Xu S., Wang J. (2015). Characterizing the Free-Energy Landscape of MDM2 Protein-Ligand Interactions by Steered Molecular Dynamics Simulations. Chem. Biol. Drug Des..

[39] Shoaib T.H., Abdelmoniem N., Mukhtar R.M., Alqhtani A.T., Alalawi A.L., Alawaji R., Althubyani M.S., Mohamed S.G.A., Mohamed G.A., Ibrahim S.R.M. (2023). Molecular Docking and Molecular Dynamics Studies Reveal the Anticancer Potential of Medicinal-Plant-Derived Lignans as MDM2-P53 Interaction Inhibitors. Molecules.

[40] Miao Y., Feher V.A., McCammon J.A. (2015). Gaussian Accelerated Molecular Dynamics: Unconstrained Enhanced Sampling and Free Energy Calculation. J. Chem. Theory Comput..

[41] Miao Y., McCammon J.A. (2017). Gaussian Accelerated Molecular Dynamics: Theory, Implementation, and Applications. Annu. Rep. Comput. Chem..

[42] Wang J., Arantes P.R., Bhattarai A., Hsu R.V., Pawnikar S., Huang Y.M., Palermo G., Miao Y. (2021). Gaussian accelerated molecular dynamics (GaMD): Principles and applications. Wiley Interdiscip. Rev. Comput. Mol. Sci..

[43] Pawnikar S., Bhattarai A., Wang J., Miao Y. (2022). Binding Analysis Using Accelerated Molecular Dynamics Simulations and Future Perspectives. Adv. Appl. Bioinform. Chem..

[44] Singh N., Li W. (2020). Absolute Binding Free Energy Calculations for Highly Flexible Protein MDM2 and Its Inhibitors. Int. J. Mol. Sci..

[45] Yang F., Wang Y., Yan D., Liu Z., Wei B., Chen J., He W. (2023). Binding Mechanism of Inhibitors to Heat Shock Protein 90 Investigated by Multiple Independent Molecular Dynamics Simulations and Prediction of Binding Free Energy. Molecules.

[46] Chen J., Wang X., Pang L., Zhang J.Z.H., Zhu T. (2019). Effect of mutations on binding of ligands to guanine riboswitch probed by free energy perturbation and molecular dynamics simulations. Nucleic Acids Res..

[47] Li M., Cong Y., Li Y., Zhong S., Wang R., Li H., Duan L. (2019). Insight Into the Binding Mechanism of p53/pDIQ-MDMX/MDM2 With the Interaction Entropy Method. Front. Chem..

[48] Miao Y., McCammon J.A. (2016). Graded activation and free energy landscapes of a muscarinic G-protein-coupled receptor. Proc. Natl. Acad. Sci. USA.

[49] Chen J., Zeng Q., Wang W., Sun H., Hu G. (2022). Decoding the Identification Mechanism of an SAM-III Riboswitch on Ligands through Multiple Independent Gaussian-Accelerated Molecular Dynamics Simulations. J. Chem. Inf. Model..

[50] Wang J., Miao Y. (2020). Peptide Gaussian accelerated molecular dynamics (Pep-GaMD): Enhanced sampling and free energy and kinetics calculations of peptide binding. J. Chem. Phys..

[51] Miao Y., Bhattarai A., Wang J. (2020). Ligand Gaussian Accelerated Molecular Dynamics (LiGaMD): Characterization of Ligand Binding Thermodynamics and Kinetics. J. Chem. Theory Comput..

[52] N S.D., Shivakumar, Kumar D.U., Ghate S.D., Dixit S.R., Awasthi A., Revanasiddappa B.C. (2023). Benzothiazole derivatives as p53-MDM2 inhibitors: In-silico design, ADMET predictions, molecular docking, MM-GBSA Assay, MD simulations studies. J. Biomol. Struct. Dyn..

[53] Dokainish H.M., Sugita Y. (2020). Exploring Large Domain Motions in Proteins Using Atomistic Molecular Dynamics with Enhanced Conformational Sampling. Int. J. Mol. Sci..

[54] Plante A., Shore D.M., Morra G., Khelashvili G., Weinstein H. (2019). A Machine Learning Approach for the Discovery of Ligand-Specific Functional Mechanisms of GPCRs. Molecules.

[55] Plante A., Weinstein H. (2021). Ligand-Dependent Conformational Transitions in Molecular Dynamics Trajectories of GPCRs Revealed by a New Machine Learning Rare Event Detection Protocol. Molecules.

[56] Wang J., Yang W., Zhao L., Wei B., Chen J. (2024). Binding Mechanism of Inhibitors to BRD4 and BRD9 Decoded by Multiple Independent Molecular Dynamics Simulations and Deep Learning. Molecules.

[57] Degiacomi M.T. (2019). Coupling Molecular Dynamics and Deep Learning to Mine Protein Conformational Space. Structure.

[58] Sun Y., Jiao Y., Shi C., Zhang Y. (2022). Deep learning-based molecular dynamics simulation for structure-based drug design against SARS-CoV-2. Comput. Struct. Biotechnol. J..

[59] Do H.N., Wang J., Bhattarai A., Miao Y. (2022). GLOW: A Workflow Integrating Gaussian-Accelerated Molecular Dynamics and Deep Learning for Free Energy Profiling. J. Chem. Theory Comput..

[60] Do H.N., Wang J., Miao Y. (2023). Deep Learning Dynamic Allostery of G-Protein-Coupled Receptors. JACS Au.

[61] Chen J., Wang J., Yang W., Zhao L., Zhao J., Hu G. (2024). Molecular Mechanism of Phosphorylation-Mediated Impacts on the Conformation Dynamics of GTP-Bound KRAS Probed by GaMD Trajectory-Based Deep Learning. Molecules.

[62] Zhao L., Wang J., Yang W., Zhao K., Sun Q., Chen J. (2024). Unveiling Conformational States of CDK6 Caused by Binding of Vcyclin Protein and Inhibitor by Combining Gaussian Accelerated Molecular Dynamics and Deep Learning. Molecules.

[63] Genheden S., Ryde U. (2015). The MM/PBSA and MM/GBSA methods to estimate ligand-binding affinities. Expert Opin. Drug Discov..

[64] Wang E., Sun H., Wang J., Wang Z., Liu H., Zhang J.Z.H., Hou T. (2019). End-Point Binding Free Energy Calculation with MM/PBSA and MM/GBSA: Strategies and Applications in Drug Design. Chem. Rev..

[65] Molani F., Webb S., Cho A.E. (2023). Combining QM/MM Calculations with Classical Mining Minima to Predict Protein-Ligand Binding Free Energy. J. Chem. Inf. Model..

[66] Naïm M., Bhat S., Rankin K.N., Dennis S., Chowdhury S.F., Siddiqi I., Drabik P., Sulea T., Bayly C.I., Jakalian A. (2007). Solvated interaction energy (SIE) for scoring protein-ligand binding affinities. 1. Exploring the parameter space. J. Chem. Inf. Model..

[67] Popowicz G.M., Czarna A., Wolf S., Wang K., Wang W., Dömling A., Holak T.A. (2010). Structures of low molecular weight inhibitors bound to MDMX and MDM2 reveal new approaches for p53-MDMX/MDM2 antagonist drug discovery. Cell Cycle.

[68] Michelsen K., Jordan J.B., Lewis J., Long A.M., Yang E., Rew Y., Zhou J., Yakowec P., Schnier P.D., Huang X. (2012). Ordering of the N-terminus of human MDM2 by small molecule inhibitors. J. Am. Chem. Soc..

[69] Raghavan S.S., Iqbal S., Ayyadurai N., Gunasekaran K. (2022). Insights in the structural understanding of amyloidogenicity and mutation-led conformational dynamics of amyloid beta (Aβ) through molecular dynamics simulations and principal component analysis. J. Biomol. Struct. Dyn..

[70] Sittel F., Jain A., Stock G. (2014). Principal component analysis of molecular dynamics: On the use of Cartesian vs. internal coordinates. J. Chem. Phys..

[71] Ichiye T., Karplus M. (1991). Collective motions in proteins: A covariance analysis of atomic fluctuations in molecular dynamics and normal mode simulations. Proteins.

[72] Chen J., Wang L., Wang W., Sun H., Pang L., Bao H. (2021). Conformational transformation of switch domains in GDP/K-Ras induced by G13 mutants: An investigation through Gaussian accelerated molecular dynamics simulations and principal component analysis. Comput. Biol. Med..

[73] Humphrey W., Dalke A., Schulten K. (1996). VMD: Visual molecular dynamics. J. Mol. Graph..

[74] Kabsch W., Sander C. (1983). Dictionary of protein secondary structure: Pattern recognition of hydrogen-bonded and geometrical features. Biopolymers.

[75] Liu M., Li C., Pazgier M., Li C., Mao Y., Lv Y., Gu B., Wei G., Yuan W., Zhan C. (2010). D-peptide inhibitors of the p53-MDM2 interaction for targeted molecular therapy of malignant neoplasms. Proc. Natl. Acad. Sci. USA.

[76] Liu M., Pazgier M., Li C., Yuan W., Li C., Lu W. (2010). A left-handed solution to peptide inhibition of the p53-MDM2 interaction. Angew. Chem. Int. Ed. Engl..

[77] Strizhak A.V., Babii O., Afonin S., Bakanovich I., Pantelejevs T., Xu W., Fowler E., Eapen R., Sharma K., Platonov M.O. (2020). Diarylethene moiety as an enthalpy-entropy switch: Photoisom-erizable stapled peptides for modulating p53/MDM2 interaction. Org. Biomol. Chem..

[78] Anandakrishnan R., Aguilar B., Onufriev A.V. (2012). H++ 3.0: Automating pK prediction and the preparation of biomolecular structures for atomistic molecular modeling and simulations. Nucleic Acids Res..

[79] Case D.A., Cheatham T.E., Darden T., Gohlke H., Luo R., Merz K.M., Onufriev A., Simmerling C., Wang B., Woods R.J. (2005). The Amber biomolecular simulation programs. J. Comput. Chem..

[80] Salomon-Ferrer R., Case D.A., Walker R.C. (2013). An overview of the Amber biomolecular simulation package. WIREs Comput. Mol. Sci..

[81] Jakalian A., Jack D.B., Bayly C.I. (2002). Fast, efficient generation of high-quality atomic charges. AM1-BCC model: II. Parameterization and validation. J. Comput. Chem..

[82] Wang J., Wang W., Kollman P.A., Case D.A. (2006). Automatic atom type and bond type perception in molecular mechanical calculations. J. Mol. Graph. Model..

[83] Tian C., Kasavajhala K., Belfon K.A.A., Raguette L., Huang H., Migues A.N., Bickel J., Wang Y., Pincay J., Wu Q. (2020). ff19SB: Amino-Acid-Specific Protein Backbone Parameters Trained against Quantum Mechanics Energy Surfaces in Solution. J. Chem. Theory Comput..

[84] Wang J., Wolf R.M., Caldwell J.W., Kollman P.A., Case D.A. (2004). Development and testing of a general amber force field. J. Comput. Chem..

[85] He X., Man V.H., Yang W., Lee T.S., Wang J. (2020). A fast and high-quality charge model for the next generation general AMBER force field. J. Chem. Phys..

[86] Nayar D., Agarwal M., Chakravarty C. (2011). Comparison of Tetrahedral Order, Liquid State Anomalies, and Hydration Behavior of mTIP3P and TIP4P Water Models. J. Chem. Theory Comput..

[87] Jorgensen W.L., Chandrasekhar J., Madura J.D., Impey R.W., Klein M.L. (1983). Comparison of simple potential functions for simulating liquid water. J. Chem. Phys..

[88] Joung I.S., Cheatham T.E. (2008). Determination of alkali and halide monovalent ion parameters for use in explicitly solvated biomolecular simulations. J. Phys. Chem. B.

[89] Joung I.S., Cheatham T.E. (2009). Molecular dynamics simulations of the dynamic and energetic properties of alkali and halide ions using water-model-specific ion parameters. J. Phys. Chem. B.

[90] Miao Y., Sinko W., Pierce L., Bucher D., Walker R.C., McCammon J.A. (2014). Improved Reweighting of Accelerated Molecular Dynamics Simulations for Free Energy Calculation. J. Chem. Theory Comput..

[91] Ryckaert J.-P., Ciccotti G., Berendsen H.J.C. (1977). Numerical integration of the cartesian equations of motion of a system with constraints: Molecular dynamics of n-alkanes. J. Comput. Phys..

[92] Izaguirre J.A., Catarello D.P., Wozniak J.M., Skeel R.D. (2001). Langevin stabilization of molecular dynamics. J. Chem. Phys..

[93] Essmann U., Perera L., Berkowitz M.L., Darden T., Lee H., Pedersen L.G. (1995). A smooth particle mesh Ewald method. J. Chem. Phys..

[94] Salomon-Ferrer R., Götz A.W., Poole D., Le Grand S., Walker R.C. (2013). Routine Microsecond Molecular Dynamics Simulations with AMBER on GPUs. 2. Explicit Solvent Particle Mesh Ewald. J. Chem. Theory Comput..

[95] Götz A.W., Williamson M.J., Xu D., Poole D., Le Grand S., Walker R.C. (2012). Routine Microsecond Molecular Dynamics Simulations with AMBER on GPUs. 1. Generalized Born. J. Chem. Theory Comput..

[96] McGibbon R.T., Beauchamp K.A., Harrigan M.P., Klein C., Swails J.M., Hernández C.X., Schwantes C.R., Wang L.P., Lane T.J., Pande V.S. (2015). MDTraj: A Modern Open Library for the Analysis of Molecular Dynamics Trajectories. Biophys. J..

[97] Kotikalapudi R. (2017). Keras-Vis.

[98] Roe D.R., Cheatham T.E. (2013). PTRAJ and CPPTRAJ: Software for Processing and Analysis of Molecular Dynamics Trajectory Data. J. Chem. Theory Comput..

[99] Onufriev A., Bashford D., Case D.A. (2004). Exploring protein native states and large-scale conformational changes with a modified generalized born model. Proteins.

[100] Miller B.R., McGee T.D., Swails J.M., Homeyer N., Gohlke H., Roitberg A.E. (2012). MMPBSA.py: An Efficient Program for End-State Free Energy Calculations. J. Chem. Theory Comput..

[101] Gohlke H., Kiel C., Case D.A. (2003). Insights into protein-protein binding by binding free energy calculation and free energy decomposition for the Ras-Raf and Ras-RalGDS complexes. J. Mol. Biol..

[102] Purisima E.O. (1998). Fast summation boundary element method for calculating solvation free energies of macromolecules. J. Comput. Chem..

[103] Purisima E.O., Nilar S.H. (1995). A simple yet accurate boundary element method for continuum dielectric calculations. J. Comput. Chem..

[104] Bhat S., Purisima E.O. (2006). Molecular surface generation using a variableradius solvent probe. Proteins.

[105] Perdih A., Bren U., Solmajer T. (2009). Binding free energy calculations of N-sulphonyl-glutamic acid inhibitors of MurD ligase. J. Mol. Model..

[106] Cui Q., Sulea T., Schrag J.D., Munger C., Hung M.N., Naïm M., Cygler M., Purisima E.O. (2008). Molecular dynamics-solvated interaction energy studies of protein-protein interactions: The MP1-p14 scaffolding complex. J. Mol. Biol..

