# Supplementary material for: Insights into the Interaction Mechanisms of Peptide and Non-Peptide Inhibitors with MDM2 Using Gaussian-Accelerated Molecular Dynamics Simulations and Deep Learning
Source: Molecules. 2024 Jul 18;29(14):3377. doi: 10.3390/molecules29143377 (PMC11279683; doi:10.3390/molecules29143377)
Supplement: Supplementary file 1 [file molecules-29-03377-s001.zip › molecules-3089194-supplementary.pdf]

Insights into the Interaction Mechanisms of Peptide and Non-Peptide Inhibitors with MDM2 using Gaussian-Accelerated Molecular Dynamics Simulations and Deep Learning

Wanchun Yang *, Jian Wang, Lu Zhao and Jianzhong Chen *

School of Science, Shandong Jiaotong University, Jinan 250357, China; wangjian_lxy@sdjtu.edu.cn (J.W.); zhaolusdu@163.com (L.Z.)
* Correspondence: yangwch1982@126.com (W.Y.); chenjianzhong1970@163.com or jzchen@sdjtu.edu.cn (J.C.)

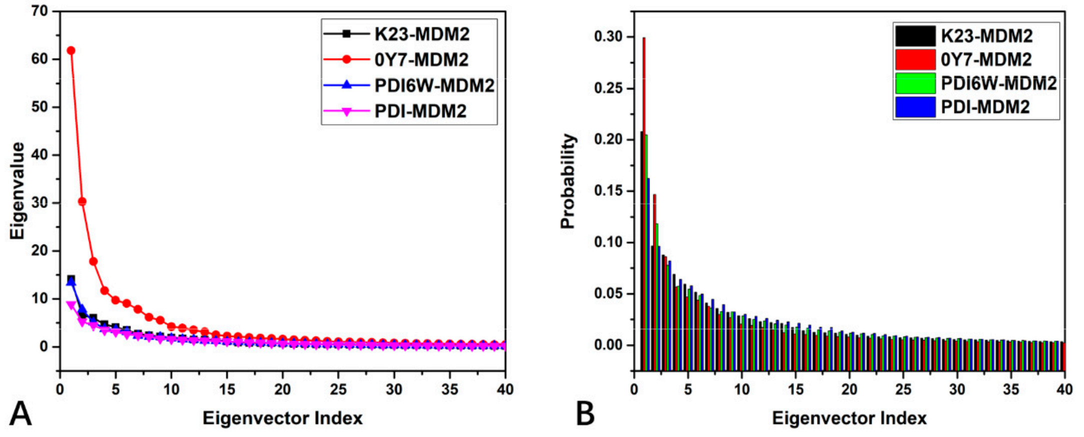

Figure S1. The eigenvalues and their corresponding proportions: (A) the function of eigenvalues over eigenvector indexes and (B) the proportions of eigenvalues over eigenvector indexes.

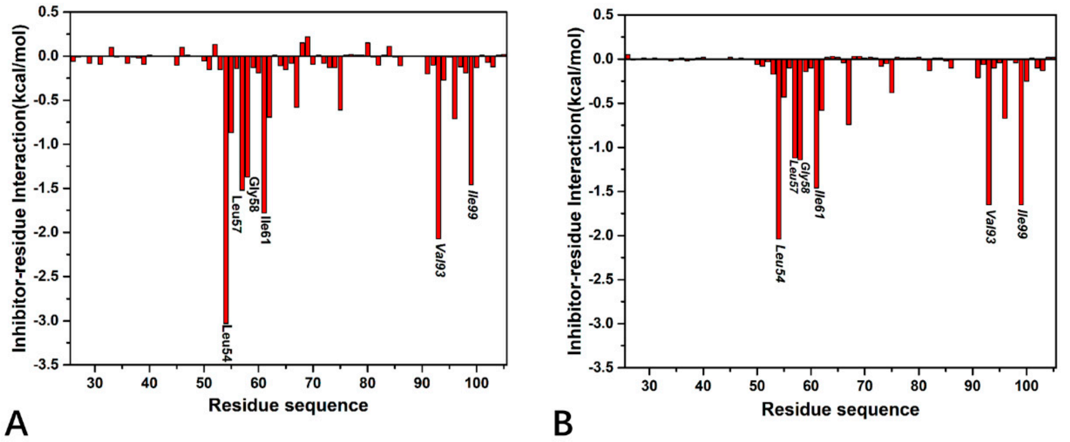

Figure S2. Interactions of inhibitors with separate residues in MDM2: (A) K23-MDM2 and (B) 0Y7-MDM2.

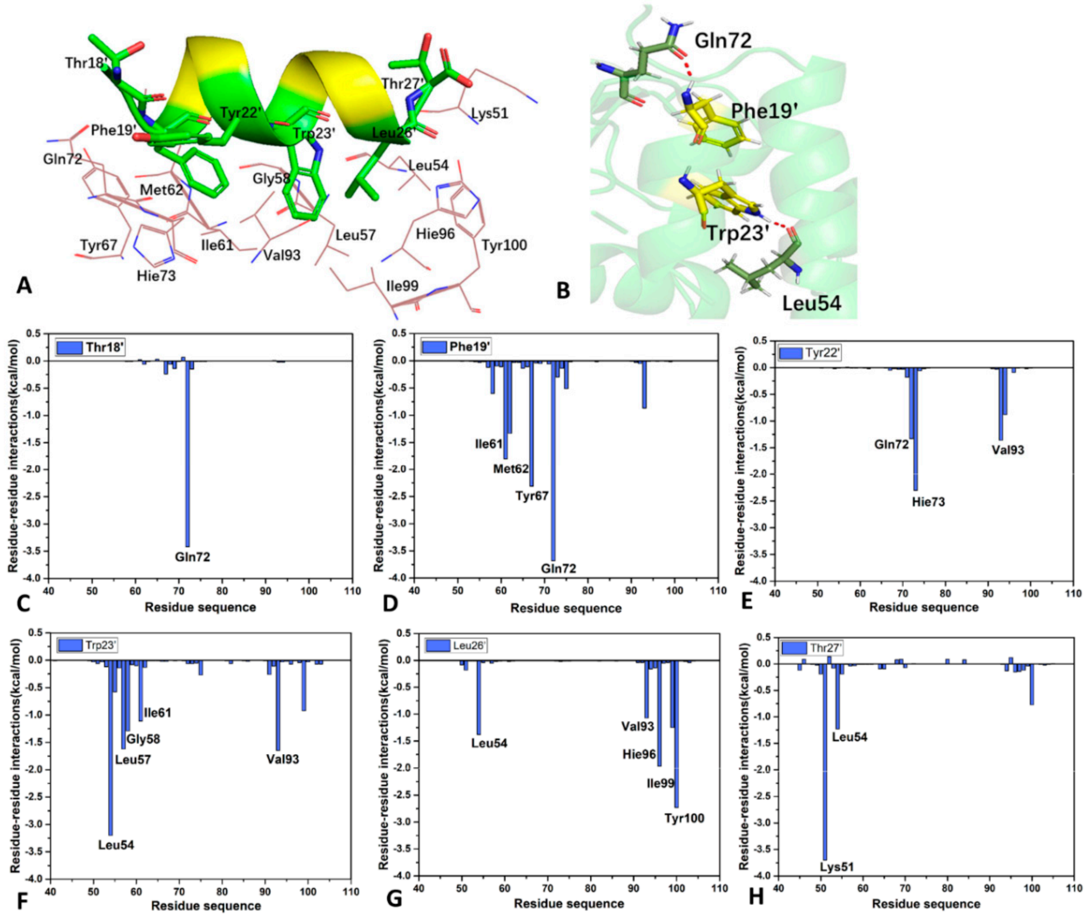

Figure S3. Interaction network of inhibitor PDI with MDM2: (A) relative geometrics of the key residues in PDl-MDM2, (B) the hydrogen bonding interactions in PDl-MDM2 and (C-H) residue-residue interaction spectrum between the inhibitor PDl and MDM2.

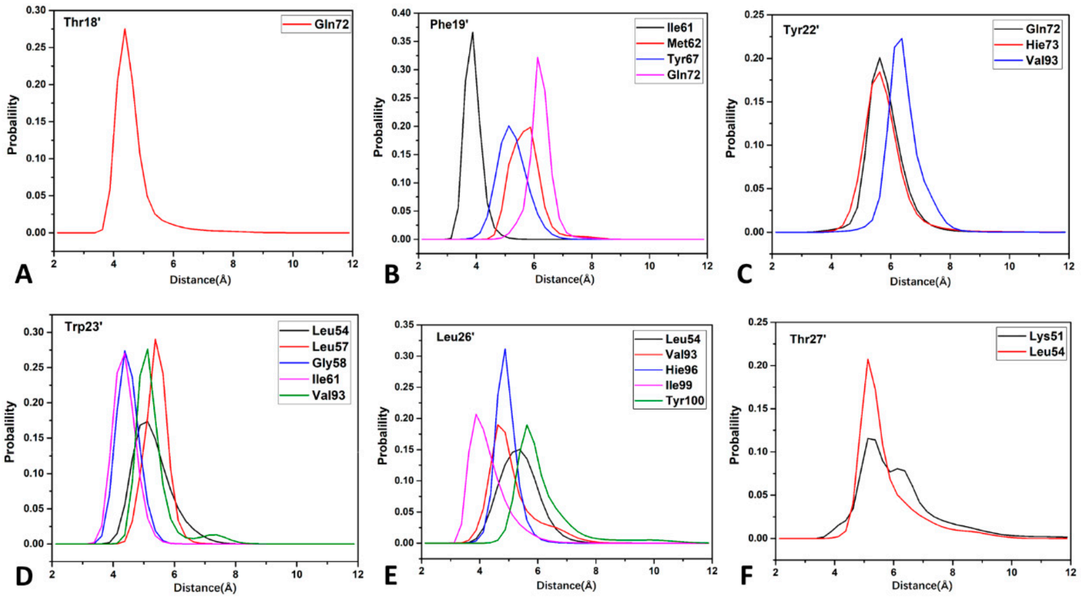

Figure S4. Probability distributions of the distances relating to key inhibitor-residue interactions for PDI-MDM2.
